# New Insights of Powder Metallurgy: Microstructure, Durability and Properties

**DOI:** 10.3390/ma16062307

**Published:** 2023-03-13

**Authors:** Jaroslav Kováčik, Anchalee Manonukul

**Affiliations:** 1 Institute of Materials and Machine Mechanics, Slovak Academy of Sciences, 845 13 Bratislava, Slovakia; 2 National Metal and Materials Technology Center (MTEC), National Sciences and Technology Development Agency (NSTDA), 114 Thailand Science Park, Klong 1, Klong Luang, Pathumthani 12120, Thailand

This Special Issue of *Materials*, entitled “New Insight of Powder Metallurgy: Microstructure, Durability and Properties”, aimed to publish original and review papers on new scientific and applied research making significant contributions to our findings and understanding of the current developments and trends in powder metallurgy (PM). Focus is placed on technology, structure, characterization, and applications in this field.

PM is a technology that includes a wide range of methods through which materials or components are produced from powders. PM processes can prevent or greatly reduce the need for further machining, drastically reduce production yield losses and often result in lower costs. PM is also used to develop unique materials that cannot be obtained by melting or shaping in other ways. The PM technologies offer flexibility in the materials, microstructure and design, as major fractions of the material remain in the solid state, and even insoluble material combinations can be employed.

The history of PM and the art of metal and ceramic sintering are intimately related. Sintering involves the production of a hard solid metal or ceramic piece from a starting powder and dates back to the Iron Age [1]. Nevertheless, recent developments in this field, such as industrial additive manufacturing (AM), selective laser sintering and other metal AM processes, represent a new category of commercially important PM applications.

PM methods are used for the manufacturing of materials where other technologies of shaping cannot be applied. A good example is the AM of materials from powders, going hand-in-hand with the production of high-purity of powders, together with the possibility of affecting the powder size and morphology. These powders determine the end properties of PM products and are highly attractive in material markets. For these reasons, in this Special Issue, articles that focus on AM preparation from powders are also included.

Papers examining sintering; process parameters; the influences of innovative methods of preparation such as electric-current-assisted sintering, microwave radiation or lasers; and sintering using concentrated solar power, fully compacted materials or porous preforms, or even foams, are also included in this Special Issue.

This SI presents high-quality articles, communications, and reviews reporting on advancements in the fascinating field of PM.

## Data Availability

Not applicable.
